# Social Representations of “Tinnitus” and “Health” among Individuals with Tinnitus Seeking Online Psychological Interventions

**DOI:** 10.3390/audiolres13020020

**Published:** 2023-03-06

**Authors:** Vinaya Manchaiah, Srikanth Chundu, Pierre Ratinaud, Gerhard Andersson, Eldre W. Beukes

**Affiliations:** 1 Department of Otolaryngology–Head and Neck Surgery, University of Colorado School of Medicine, Aurora, CO 80045, USA; 2 UCHealth Hearing and Balance, University of Colorado Hospital, Aurora, CO 80045, USA; 3 Virtual Hearing Lab, Collaborative Initiative between University of Colorado School of Medicine and University of Pretoria, Aurora, CO 80045, USA; 4 Department of Speech-Language Pathology and Audiology, University of Pretoria, Pretoria 0002, South Africa; 5 Department of Speech and Hearing, Manipal College of Health Professions, Manipal Academy of Higher Education, Manipal 576104, India; 6 Vision and Hearing Sciences Research Group, School of Psychology and Sport Science, Anglia Ruskin University, Cambridge CB1 1PT, UK; 7 Laboratoire d’Études et de Recherches Appliquées en Sciences Sociales (LERASS), University of Toulouse, 31000 Toulouse, France; 8 Department of Behavioral Sciences and Learning, Linköping University, 58183 Linköping, Sweden; 9 Department of Biomedical and Clinical Sciences, Linköping University, 58183 Linköping, Sweden; 10 Department of Clinical Neuroscience, Division of Psychiatry, Karolinska Institute, 17177 Stockholm, Sweden

**Keywords:** tinnitus, health, social representations, attitude, free association

## Abstract

(1) Objective: Social representations theory (SRT) is a body of theory within social psychology concerned with how individuals, groups, and communities collectively make sense of socially relevant or problematic issues, ideas, and practices. SRT has been increasingly sued in the area of health and disability. The current study examined the social representations of “tinnitus” and “health” among individuals with tinnitus who are seeking online psychological interventions. (2) Materials/Method: The data were gathered using a free association task about their “tinnitus” and “health” from 399 individuals with tinnitus. The data were analyzed using both qualitative and quantitative analyses methods. (3) Results: The responses resulted in 39 and 30 categories respectively, for “tinnitus” and “health”. The most commonly occurring categories for tinnitus included: *descriptions of tinnitus* (18%), *annoying* (13.5%), *persistent* (8%), and *distracting* (5%). The most commonly occurring categories for health included: *content* (12%), *conditions* (8%), *active* (7%), *take control* (6%), and *overweight* (5%). The responses to tinnitus had predominantly negative connotations (i.e., 76.9%) whereas a larger proportion of responses toward their health was related to positive connotations (i.e., 46.4%). These frequently occurring items were also dominant in similarities analysis. Prototypical analysis of tinnitus responses identified categories *horrible* and *bothersome* to be key items in the central zone. The categories in central zone of health responses included: *content*, *active*, *healthy*, *grateful,* and *overweight*. (4) Conclusions: Individuals with tinnitus have very negative view of their tinnitus impacting their psychological status. Tinnitus management should focus on reducing the negative associations toward their tinnitus and strengthen the positive aspects related to their general health.

## 1. Introduction

Tinnitus is a common condition and is experienced by 10–15% of the adult population, although only a small percentage (roughly 2%) are severely annoyed by this condition [1]. While various factors including pathophysiological aspects may contribute to the experience, it is still unclear why some people state that tinnitus does not interfere with everyday life whereas others find it extremely debilitating [2,3,4]. One possible explanation for this comes from the psychological model of tinnitus which suggests that cognitive factors play a crucial role in tinnitus experiences [5]. Moreover, that tinnitus should be viewed as a multidimensional symptom which can only be understood fully from the biopsychosocial perspective [6]. For these reasons, all theories including biological underpinnings, psychological mechanisms, as well as social aspects need to be examined to better understand the tinnitus experience [3,4,7].

In clinical settings, questioning about tinnitus experiences almost always carry a negative connotation. For example, clinicians may ask their tinnitus patients to make a list of difficulties or life changes they have experienced because of their tinnitus [6]. Naturally, such questions generally produce negative responses due to the resulting difficulties related to bothered tinnitus. However, in some studies individuals with tinnitus have also reported positive experiences as a result of their tinnitus when probed specifically about these [8,9]. Such reports of positive experiences are not unique to tinnitus as a condition but can be seen in various chronic conditions including cancer, hearing loss, and balance problems [10]. These findings demonstrate that a broader theoretical framework is needed to examine the wider consequences of tinnitus without prompting them toward either positive or negative experiences.

There are various health behavior theories such as Health Belief Model or Theory of Reasoned Action which examine which one provides an understanding of the health condition from an individual level [11]. However, in recent years the Social Representations Theory (SRT) has become more popular in disability studies to get an understanding of the health condition from a social or a collective level [12]. Moreover, the methodology used in SRT tend to examine the issue in question in a more neutral way [13]. For example, often the participants are asked to report what comes to mind when they think about a phenomenon (e.g., a health condition). Such a spontaneous response without providing any cue is likely to provide a better understanding of shared ideas and values people have within the sociocultural context [12]. Hence, SRT could help better understand how individuals with tinnitus think about their condition in a more neutral way.

The concept of SRT was formulated by Serge Moscovici in 1961 who described “social representations” as “social psychological approach articulating individual thinking and feeling with collective interaction and communication” [13]. Social representations are formed during our daily interactions and communications, and they define how we interact with each other. The name “social” indicates that representations created are social and accommodate multifactorial factors including cultural, economic, political, and religious beliefs [14]. The key difference between this and the psychological theories is that the SRT tends to provide an understanding of collective thinking, whereas the psychological theories often target the attitude of the individual. For this reason, the SRT is seen as an alternative theoretical framework in understanding health and disability and has been increasingly used in disability literature [12]. Although SRT has been subjected to its critiques due to its broad nature as well as for not having a hypothesis driven approach [15], we believe that there is value in applying SRT to examine various chronic conditions such as tinnitus to get a multidimensional understanding.

The current exploratory study was aimed at understanding the social representations of “tinnitus” and “health” in individuals with tinnitus who are seeking online psychological treatment. This group comprises those who are bothered by their tinnitus and are looking for clinical management. For this reason, their perspectives may be more important when planning rehabilitation. In addition, in this study we included the exploration about their general health in addition to their tinnitus to see if there is any relation between these two concepts of interest.

## 2. Method

### 2.1. Study Design and Ethical Considerations

The current study used an exploratory cross-sectional survey design. Individuals with tinnitus who were seeking online psychological treatment (i.e., internet-based cognitive behavioral therapy for tinnitus; ICBT) by registering to three separate clinical trials (ClinicalTrials.gov registration numbers NCT04004260, NCT04335812) were included [16,17,18]. Ethical approval (IRB-FY17-209 and IRB-FY20-200-1) was obtained from the Institutional Review Board at Lamar University, Beaumont, TX, USA.

### 2.2. Data Collection

The data were gathered pre-intervention using a secure platform for online questionnaires consisting of demographic and tinnitus-related questions, standardized patient-reported outcome measures (PROMs), and response to free association tasks.

The standardized PROMs included Tinnitus Functional Index (TFI [19]) as a measure of tinnitus distress, Generalized Anxiety Disorder-7 (GAD7 [20]) as a measure of anxiety, Patient Health Questionnaire-9 (PHQ-9 [21]) as a measure of depression, Insomnia Severity Index (ISI [22]) as a measure of insomnia, and the EQ-5D-5L VAS scale [23] as a measure of general health-related quality of life. In addition, participants were asked to provide answers to a free association task about tinnitus and health. In this task, participants were prompted to come up with five words or phrases that spontaneously come to their mind when they think about their tinnitus and to write them in the order or importance (i.e., most important being the first word/phrase and the least important being the fifth word/phrase). In the next step, they were asked to consider each of the words or phrases they came up with and indicate if it had positive, neutral, or negative connotations. The same task was repeated when they thought about their health as the object of interest. The free association task described above has been commonly used in gathering data for social representation studies especially in the area of health and disability [24,25,26,27,28,29,30]. Due to the spontaneous nature of the free association task, it is suggested that this will help tap into the sematic universe of participants and minimize social desirability bias [31].

### 2.3. Participants

Of the 440 people who initiated the registration for ICBT clinical trials, 41 did not complete the questionnaire relevant to this study and were excluded; the remaining 399 participants were included. The demographic and clinical variables are shown in Table 1. The participants were well balance for gender with 52% being female (Table 1). Their mean age was 55 years, and they had an average duration of tinnitus of 12 years. The majority were educated with a college or a university degree (89%), and performed skilled or professional work (61%). The level of tinnitus distress indicated severe tinnitus indicating the need for a tinnitus intervention.

## 3. Data Analysis

The data were analyzed using various qualitative (content analysis) and quantitative (i.e., Chi square analysis, similarities analysis, and prototypical analysis) methods which are detailed below. However, a more detailed description of these methods can be found in our earlier publications [12,26,27]. The similarities and prototypical analysis were conducted using the open-source text analysis software, IraMuTeQ. It is noteworthy that the studies on social representations generally apply multiple types of analyses to get multidimensional understanding of the data [12].

***Content analysis:*** The response to free-association task were analyzed using qualitative content analysis [32]. This involved the grouping of similar words (e.g., anger, rage, hate) into a category (e.g., angering).

***Chi square analysis:*** The frequency of positive, neutral, and negative connotations for tinnitus and health were counted. Chi square analysis (3 × 2) was performed to examine the association between connotations and the object of interest.

***Similarities analysis:*** The similarities analysis involves understanding most important categories and there interrelation to each other. This analysis was based on the mathematical graph theory [33], and the output is presented in a two-dimensional graph with nodes and connections (i.e., Jaccard index). Each node represents a category, and the size of the node represents the frequency of the category with bigger nodes representing higher frequency. The lines linking the nodes represent the interconnections between categories and the thickness of the line indicate strength of these connections. This analysis only considers the categories and its association, but not the ranking of each word or association listed.

***Prototypical analysis:*** This analysis considers both frequency as well as importance ratings (or ranking) of words or expressions provided in the free association task and offers content and structure of social representations. The results are presented in a 2 × 2 matrix with four elements [34]. The “central zone” of the most important of these is represented by the most frequently occurring as well as most important categories. The “first peripheral zone” includes the second most important categories which are the most frequently occurring categories but less important in terms of its ranking. The “second peripheral zone” represents the categories that are less frequent as well as less important in terms of ranking in the free association task. Finally, the “contrasted elements” include the low frequency categories but rated as most important in terms of its rankings. Generally, the categories in the central zone are considered as the core of social representations and are very stable [34]. On the other hand, the peripheral elements are considered less stable as they vary across people and environments. The contrasted elements highlight a sub-group of population that has a different priority than the general (majority) population.

## 4. Results

### 4.1. Content Analysis

Table 2 and Table 3 provide a summary of key categories and the frequency for responses related to “tinnitus” and “health”, respectively. Using the content analysis, similar responses (i.e., words or expressions) were grouped to produce a small number of meaningful categories. The responses for “tinnitus” resulted in 39 unique categories. Out of these the most commonly occurring categories for “tinnitus” were: a term describing the nature of the tinnitus (18%), annoying (13.5%), persistent (8%), distracting (5%), and distressing (4%). The responses for health resulted in 30 unique categories. The most commonly occurring categories for “health” included: content (12%), conditions (8%), active (7%), take control (6%), overweight (5%), and distressing (4%). These frequently occurring items were also dominant in similarities analysis.

### 4.2. Distribution of Connotations

**Figure 1:**
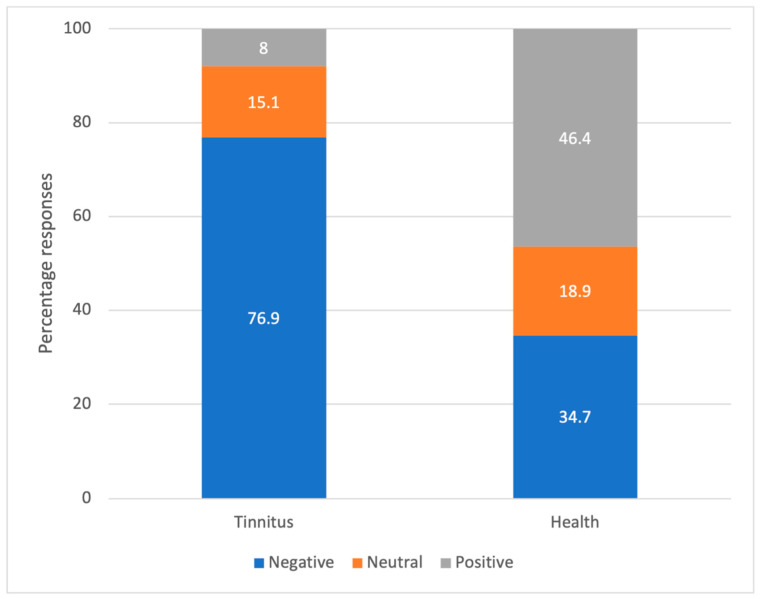
Percentages of associations ranked positive, neutral, and negative for responses to free association task about tinnitus and health.

Figure 1 presents the distribution of positive, negative, and neutral connotations associated with the response to free association task about “tinnitus” and “health”. The responses to “tinnitus” were predominantly negative connotations (i.e., 76.9%) with only a small percentage (8%) of responses being positive. In comparison, a larger proportion of responses toward their “health” had positive connotations (i.e., 46.4%), although 34.7% and 18.9% of responses were positive and neutral, respectively. The chi square analysis suggested significant association between connotations and the response category of either tinnitus or health (Chi square = 859, *p* < 0.001).

### 4.3. Similarities Analysis

**Figure 2:**
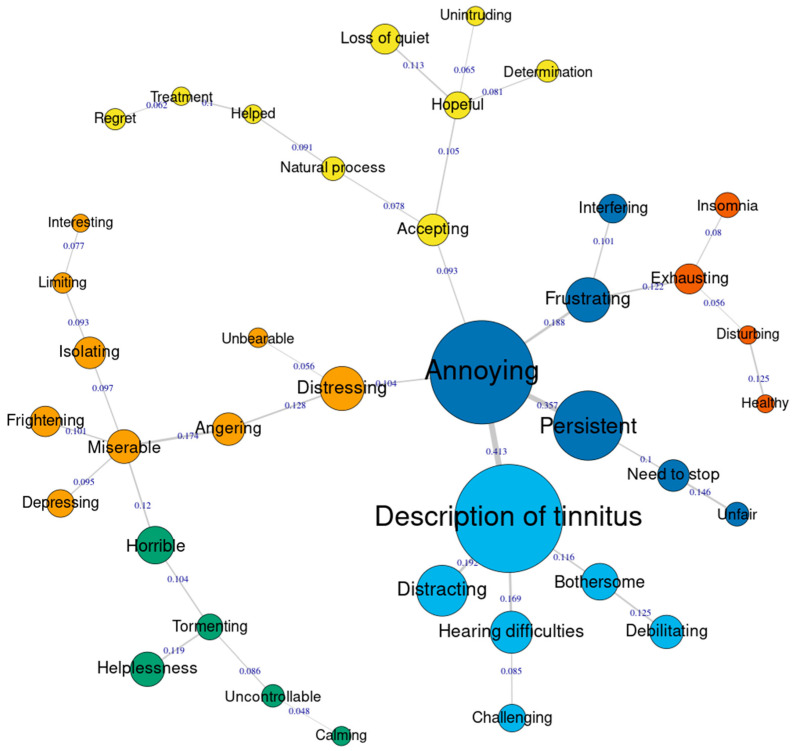
Similarities analysis for tinnitus responses.

**Figure 3:**
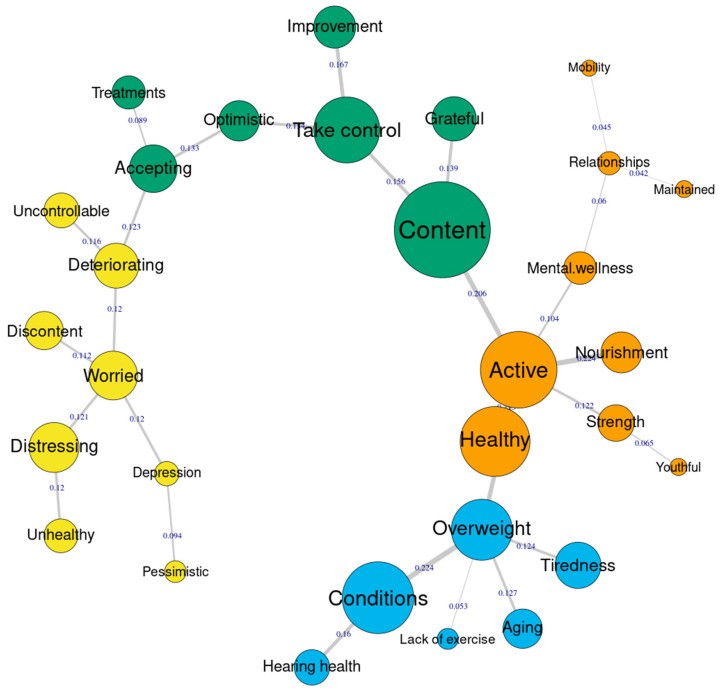
Similarities analysis index for health responses.

Figure 2 and Figure 3 present the similarities analysis results of “tinnitus” and “health”, respectively in a matric tree index. Here, the size of the nodes represents the frequency of the category and the thickness of the line connecting the nodes suggest how strongly one category is related to the other (co-occurrence). In other words, how often two categories were reported by the same person. For “tinnitus” (Figure 2), five dominant nodes were identified, namely description of tinnitus, annoying, persistent, distressing, and frustrating. Interestingly, some nodes were connected to category *accepting*, although they were not dominant. The category “annoying” was a central node, connecting the categories frustrating, persistent, deception of tinnitus, and distressing. This means, people who reported annoying also reported other categories that are connected to this. In a similar way, people who reported accepting also reported responses such as natural process or hopeful. For “health” (Figure 3), the dominant nodes include the categories content, take control, active, healthily, overweight, and conditions. For health the category “content” seems to be the central node connecting other categories. Although majority of the responses appear to be positive, the nodes related to categories overweight, condition, deteriorating, and worried appear to be negative.

### 4.4. Prototypical Analysis

As the similarities analysis only considers the frequency and inter-connectedness but not the ranking of responses based on importance, further analysis was conducted to examine the most important associations based on ranking as well as frequency. Table 4 and Table 5 presents the results of prototypical analyses for “tinnitus” and “health” responses, respectively. The central zone of “tinnitus” included four categories that include description of tinnitus, annoying, horrible, and bothersome. However, the categories in the central zone of health responses included: content, active, healthily, overweight, and grateful. These are the items that are most important elements based on their ranking as well as frequency. Although categories persistent, distracting, and distressing were frequently reported, they are least important compared to categories horrible and bothersome. These elements tend to be most stable and are difficult to change. It is interesting to note that the majority of the categories related to “tinnitus” were in second periphery (see Table 4), indicating that they are least frequent as well as low ranking. Moreover, no categories that were positive in “tinnitus” responses appeared in the central zone, although some positive categories were found in the first periphery and contrasted elements indicating that the positive responses (if any) were ranked lower in relation to their tinnitus. On the other hand, there was a good distribution of categories across the matrix. Moreover, it is interesting to note that the elements of the central zone of “health” were generally positive (i.e., content, being active, healthy, grateful) suggesting that these individuals had positive opinion or outlook about their general health. Categories such as conditions, take control, and distressing were frequently reported by respondents, but when combining both frequency and rank they are in the first periphery (most frequent but least important) rather than the central zone.

## 5. Discussion

The current study examined the representations of “tinnitus” and “health” among individuals with tinnitus using a new theoretical perspective. The responses were analyzed using a range of qualitative and quantitative analyses methods to get a multidimensional understanding of the data. The following sections highlight some key findings and how these are related to the existing literature.

When examining the words or expressions provided by participants about their “tinnitus”, a large proportions of these responses (13.5%) focused on descriptions of tinnitus (e.g., a high-pitched or ringing sound). Moreover, individuals also presented responses about tinnitus being persistent (8%), distracting (5%), and distressing (4%). These results are not surprising as constant awareness of sound as well as its impact [35,36]. The common categories about “health” responses included content (12%), conditions (8%), being active (7%), take control (6%), overweight (5%), and distressing (4%) which is suggesting that individuals in the current study sample have more positive outlook about their general health. Moreover, when examining the connotations associated with the responses, the positive and negative views toward their “tinnitus” and “health” was more evident. For instance, tinnitus was associated with negative connotations whereas “health” was associated with mixture of positive and negative associations. These results suggest that there was not much commonality on how individuals with tinnitus think about their tinnitus and their general health. Studies on clinical populations as well as examination of social media data on tinnitus has highlighted the negative association with tinnitus [37,38]. However, interestingly a small percentage of responses about tinnitus were positive which were related to accepting the condition, thinking that this is natural process, and being hopeful. A few recent studies have reported individuals with tinnitus reporting positive experiences as a result of their condition [8,9]. This may demonstrate their temperament and acceptance of the condition. Taken together, these findings have important therapeutic implications as reducing negative associations and strengthening positive associations should be the goal of psychological management of tinnitus.

The similarities analysis provided some useful insights into what categories are related. Interesting to note that categories that were positive and/or negative were grouped together. In other words, when respondents provide some positive (or negative) responses, they are likely to be providing similar responses. The category *annoying* was reported by respondents who also reported other negatively associated categories such as frustrating, persistent, and distressing indicating that tinnitus can lead to negative emotions (add some supporting literature). In the current study, a small subgroup of respondents who reported category annoying also reported accepting highlighting that respondents may be positive if they accept tinnitus (literature). Moreover, prototypical association identified an important finding suggesting that although individuals with tinnitus have very negative elements in their central zone (e.g., horrible, bothersome), they have surprisingly positive elements about “health” in the central zone (e.g., content, active, healthy, grateful). In other words, individuals with tinnitus do not have much commonality regarding how they think about their health and about their tinnitus. There is growing interest in examining the relationship between general health and tinnitus [39], hence this area needs to be further explored in future studies.

### 5.1. Theoritical and Practice Implications

As highlighted earlier, a biopsychosocial perspective is needed to get a multidimensional perspective of tinnitus [5,6] as it is a highly heterogeneous condition [16,17]. SRT provides a new theoretical framework to understand tinnitus. Using free association task has several advantages as it is more neutral and also open-ended which elicits responses from participants that truly matter to them. Moreover, as the social representations are influenced by elements such as cultural, economic, political, and religious beliefs [14], these representations provide insights that are beyond the understanding of tinnitus as a body function as it is often addressed clinically. The study results also have practice implications in terms of public health. Proponents of SRT suggest that media has a big role to play in the way social representations are create and changed [12]. Hence, developing public health campaigns through media especially through digital and social media may help create more positive views and association about tinnitus. This can be seen as a first step in addressing the impact of tinnitus in a population level.

### 5.2. Study Limitations and Future Directions 

The current study is to our knowledge the first to examine the social representations of tinnitus. There are limitations that need to be taken into account. The study sample included only those with bothersome tinnitus who were seeking psychological interventions. Moreover, as the participants self-selected to participate in the study, sampling bias may exist. Characteristics of study sample suggest that the study included a heterogeneous sample. However, majority of the participants (>80%) had consulted hearing healthcare professionals before enrolling in the study which may suggest that this sample is close to the clinical tinnitus population. Nevertheless, the study results should be considered exploratory and should not be generalized to all individuals with tinnitus. There is a tremendous scope for further research in this area. More specifically, we propose three specific questions for future research. First, the current study examined the social representations collectively for all participants. However, future studies may examine the sub-group of participants within this based their responses using cluster analysis [40,41]. Second, while it is hard to change the social representations, it is suggested that a strong intervention can influence the social representations. Future studies should examine whether psychological interventions such as cognitive behavioral therapy (CBT) for tinnitus have any bearing toward changing the social representations especially in strengthening positive associations and reducing negative associations. In addition, social representations of different sub-groups of tinnitus as well as how social support (e.g., support from friends/family, peer-support groups) may influence the social representations would be interesting to be considered in future studies.

## Figures and Tables

**Table 1:** Demographic details (n = 399).

Variable	N (%)	Mean (SD); Range
Age (in years)		54.8 (12.9); 19 to 84
Gender		
▪ Female ▪ Male	208 (52.1%) 191 (47.9%)	
Education		
▪ Less than high school ▪ High school ▪ Some college but not a degree ▪ A university degree	5 (1.2%) 41 (10.3%) 115 (28.8%) 238 (59.7%)	
Work status		
▪ Entry level or unskilled ▪ Skilled or professional work ▪ Retired ▪ Not working	13 (3.3%) 242 (60.6%) 112 (28.1%) 32 (8%)	
Tinnitus duration (in years)		11.9 (12.9); 0.08 to 70
Tinnitus severity (TFI)		53.1 (21.8); 4.4 to 100
Anxiety (GAD-7)		7.4 (5.7); 0 to 21
Depression (PHQ-9)		7.5 (6); 0 to 27
Insomnia (ISI)		11.5 (6.8); 0 to 28
Quality of life (EQ-5D-5L VAS)		74.3 (15.9); 9 to 100

**Table 2:** Percentage of categories for tinnitus.

Category (Examples)	Frequencies	
	N	%
Accepting (e.g., just deal with it, it is what it is, don’t think much)	45	2.37
Angering (e.g., anger, rage, hate)	46	2.42
Annoying (e.g., annoying, irritation, nuisance)	257	13.53
Bothersome (e.g., bothersome, bothered, bothers me)	52	2.74
Calming (e.g., relax, relief, prayer)	7	0.37
Challenging (e.g., challenge, difficult, a struggle)	28	1.47
Debilitating (e.g., disability, illness, handicap, impairing)	45	2.37
Depressing (e.g., depression, depressing, suicide)	29	1.53
Description of tinnitus (e.g., ringing, high-pitched, noise, buzz, loud)	342	18
Determination (e.g., cope, beatable with right mindset, I can overcome this)	14	0.74
Distracting (e.g., distraction, hard to concentrate, focusing	96	5.05
Distressing (e.g., overwhelming, anxiety, panic)	83	4.37
Disturbing (e.g., noise in my head, noise really bad, screaming)	5	0.26
Exhausting (e.g., exhaustion, wearing, tiresome)	35	1.84
Frightening (e.g., worry some, claustrophobic, scary)	36	1.89
Frustrating (e.g., frustrated, frustrating, maddening)	68	3.58
Healthy (e.g., healthy, relax, stability)	4	0.21
Hearing difficulties (e.g., can’t hear, hard to hear, repeat)	73	3.84
Helped (e.g., helped, helpful)	6	0.32
Helplessness (e.g., helpless, no cure, incurable)	48	2.53
Hopeful (e.g., hope, coping, find improvement)	28	1.47
Horrible (e.g., crazy, horror, horrible disease)	56	2.95
Insomnia (e.g., lack of sleep, sleep less, no sleep)	21	1.11
Interesting (e.g., Unique, variable, men get more than women)	4	0.21
Interfering (e.g., interfering, interruption, interference)	32	1.68
Isolating (e.g., isolated, no one understands, inhibitive)	40	2.11
Limiting (e.g., limiting, limits your life, limitation)	10	0.53
Loss of quiet (e.g., loss of silence, lack of peace, no quiet)	37	1.95
Miserable (e.g., sad, misery, bummer)	45	2.37
Natural process (e.g., aging, old age, old)	21	1.11
Need to stop (e.g., please stop, make it go away, when will it go away)	43	2.26
Persistent (e.g., constant, never ending, persistent)	151	7.95
Regret (e.g., regret not protecting hearing, poor choices, lesson learned)	12	0.63
Tormenting (e.g., torture, tormenting, a living hell)	27	1.42
Treatment (e.g., hearing aids, ear surgery, therapies that help	5	0.26
Unbearable (e.g., exasperating, unbearable, uncomfortable)	8	0.42
Uncontrollable (e.g., uncontrollable, lack of control, losing control)	15	0.79
Unfair (e.g., why me? unfair, not fair)	18	0.95
Unintruding (e.g., background noise masking, masked, in the background)	8	0.42

**Table 3:** Percentage of categories for health.

Category	Frequencies	
	N	%
Accepting (e.g., I’ll live with it, could be worse, acceptable)	69	3.67
Active (e.g., exercise, fitness, staying active, walking, workout)	132	7.02
Aging (e.g., aging, getting old, old age)	48	2.55
Conditions (diabetes, low blood pressure, back discomfort	150	7.97
Content (e.g., cheerfulness, good, somewhat satisfied))	223	11.86
Depression (e.g., depressed, sad, depressing)	22	1.17
Deteriorating (e.g., deteriorating, weakened, loss of strength)	64	3.4
Discontent (e.g., lackluster, room for improvement, been better)	49	2.6
Distressing (e.g., chronic pain, distressing, frustrating)	77	4.09
Grateful (e.g., blessed, important, thankful)	68	3.62
Healthy (e.g., overall healthy, better than most, balanced)	126	6.7
Hearing health (e.g., tinnitus, ear infections, hearing loss)	43	2.29
Improvement (e.g., better, improved, improving)	62	3.3
Lack of exercise (e.g., lazy, need to exercise, not enough exercise)	15	0.8
Maintained (e.g., take care of self, be good to self, attentive)	7	0.37
Mental wellness (e.g., easy to relax, rested, peace)	42	2.23
Mobility (e.g., mobility, staying flexible, mobile)	5	0.27
Nourishment (e.g., diet, food, keto)	51	2.71
Optimistic (e.g., hopeful, positive attitude to health, capable)	54	2.87
Overweight (e.g., overweight, obese, need to lose weight)	95	5.05
Pessimistic (e.g., it will never get better, no cure or treatment)	17	0.9
Relationships (e.g., family, beloved, friends)	25	1.33
Strength (e.g., strong, endurance, getting strong)	44	2.34
Take control (e.g., take care of myself, in my control, working on it)	110	5.85
Tiredness (e.g., tired, fatigue, no sleep_	69	3.67
Treatments (e.g., holistic, doctor, medication)	44	2.34
Uncontrollable (e.g., challenging unpredictable, in flux)	49	2.6
Unhealthy (e.g., not good, unhealthy, affected)	40	2.13
Worried (e.g., uncertain, worried, scared)	74	3.93
Youthful (e.g., been told I look young, youthful for my age, young)	7	0.37

**Table 4:** Prototypical analysis of tinnitus responses.

**Central Zone** Description of tinnitus Annoying Horrible Bothersome	**First periphery** Persistent Distracting Distressing Hearing difficulties Frustrating
**Contrasted elements** Miserable Frightening Tormenting Limiting Unbearable Disturbing	**Second periphery** Helplessness Angering Debilitating Accepting Need to stop Isolating Loss of quiet Exhausting Interfering Depressing Hopeful Challenging Insomnia Natural process Unfair Uncontrollable Determination Regret Unintruding Calming Helped Treatment Interesting Healthy

**Table 5:** Prototypical analysis of health responses.

**Central Zone** Content Active Healthy Overweight Grateful	**First periphery** Conditions Take control Distressing Worried Accepting Tiredness Deteriorating
**Contrasted elements** Improvement Aging Strength Unhealthy Lack of exercise Maintained Mobility	**Second periphery** Optimistic Nourishment Uncontrollable Discontent Treatments Hearing health Mental wellness Relationships Depression Pessimistic Youthful

## Data Availability

The data that support the findings of this study are openly available in Figshare at http://doi.org/10.6084/m9.figshare.13681924 (accessed on 15 December 2022).
